# The Longer-Term Psychosocial Development of Adolescents: Child Development Accounts and the Role of Mentoring

**DOI:** 10.3389/fped.2018.00147

**Published:** 2018-05-23

**Authors:** Ko Ling Chan, Camilla Kin Ming Lo, Frederick Ka Wing Ho, Shimin Zhu, Simon Man Kin Lai, Patrick Ip

**Affiliations:** ^1^Department of Applied Social Sciences, The Hong Kong Polytechnic University, Kowloon, Hong Kong; ^2^Department of Paediatrics and Adolescent Medicine, Li Ka Shing Faculty of Medicine, The University of Hong Kong, Pokfulam, Hong Kong

**Keywords:** poverty, child development accounts, health-related quality of life, mentoring, adolescent health, social support

## Abstract

**Objective:** To examine the long-term development of adolescents who participated in the Child Development Fund (CDF), which was a community intervention that consisted of Child Development Accounts (CDAs) and mentorship components.

**Design:** This was an evaluative study of the CDF community intervention and was conducted between January and June 2016 in Hong Kong.

**Participants:** A total of 902 adolescents from low socioeconomic backgrounds participated in this study (552 in the CDF and 350 in the comparison group). All CDF participants completed the 3-year CDF program between 2011 and 2015.

**Main outcome measures:** We assessed different developmental aspects of the adolescents, including health in terms of health-related quality of life; behavioral problems; attitude in terms of hope; cognitive capacity in terms of schooling; and social aspects in terms of social support.

**Results:** Compared to the non-participants, the CDF participants appeared to have fewer behavioral problems, higher levels of perceived social support, higher levels of hope, better understanding of academic subjects, higher levels of motivation to study, fewer school withdrawal behaviors, and better quality of life related to social functioning. The male gender moderated the program's effect on hope. Results also show that higher levels of mentorship quality moderated the program's effect on social support, hope, self-perceived understanding of academic subjects, and motivation to study.

**Conclusion:** Adolescents who participated in the CDF program appeared to perform better than the non-participants in regard to behavioral, academic, attitudinal, and social aspects. Good quality of mentorship had a positive influence on the program's effects. The CDF appears to be a promising program offering long-term and multi-dimensional benefits to participants.

## Introduction

In developed countries, such as the U.S. and the U.K., 41% and 30% of children, respectively, live in low-income families (1, 2). Children growing up in socioeconomically disadvantaged environments suffer from impeded physical health, cognitive development, and behavioral health (3, 4) through multiple processes, including lack of cognitive stimulation and learning opportunities, inadequate parenting practices, poor nutrition, cumulative socioenvironmental risks, and stress (5, 6). Investing in disadvantaged children during early stages of their development generates greater benefits for the children and their later lives, as well as in regard to lessening the economic burden to society (7).

Sherraden (8) argues that anti-poverty policies in terms of safety nets and cash assistance are insufficient for children's long-term development. The author proposed Child Development Accounts (CDAs), which constitute an asset-building approach that encourages asset accumulation for children, starting at birth. CDAs have been implemented in the U.S., the U.K., Canada, Korea, and Singapore, and have been successful in helping low-income households to accumulate savings for children's education and developmental purposes (9). However, little is known about the impacts of CDAs on children's longer-term development (10). Furthermore, it has been conceptualized that CDAs may bring about non-financial impacts on children through enhancing their well-being, academic achievement, and future orientation (11) yet, only a few empirical studies have demonstrated the positive effects of CDAs on children's social-emotional development and academic achievement, and their parents' expectations for their education and the reduction of maternal depression (12–15).

Supplemental intervention for CDAs may be able to help children and adolescents overcome multiple challenges (11). As shown by a meta-analytic study, mentoring has been widely implemented as a strategy to aid positive youth development and has shown positive influences on adolescents' prosocial behaviors, attitudes toward school and careers, interpersonal relationships, and motivation (16). The support and guidance from mentors may compensate for the lack of social resources available to disadvantaged children (17) which may in turn empower them to deal with life's challenges. However, whether mentoring augments the effects of CDAs is largely unknown.

**Figure 1:**
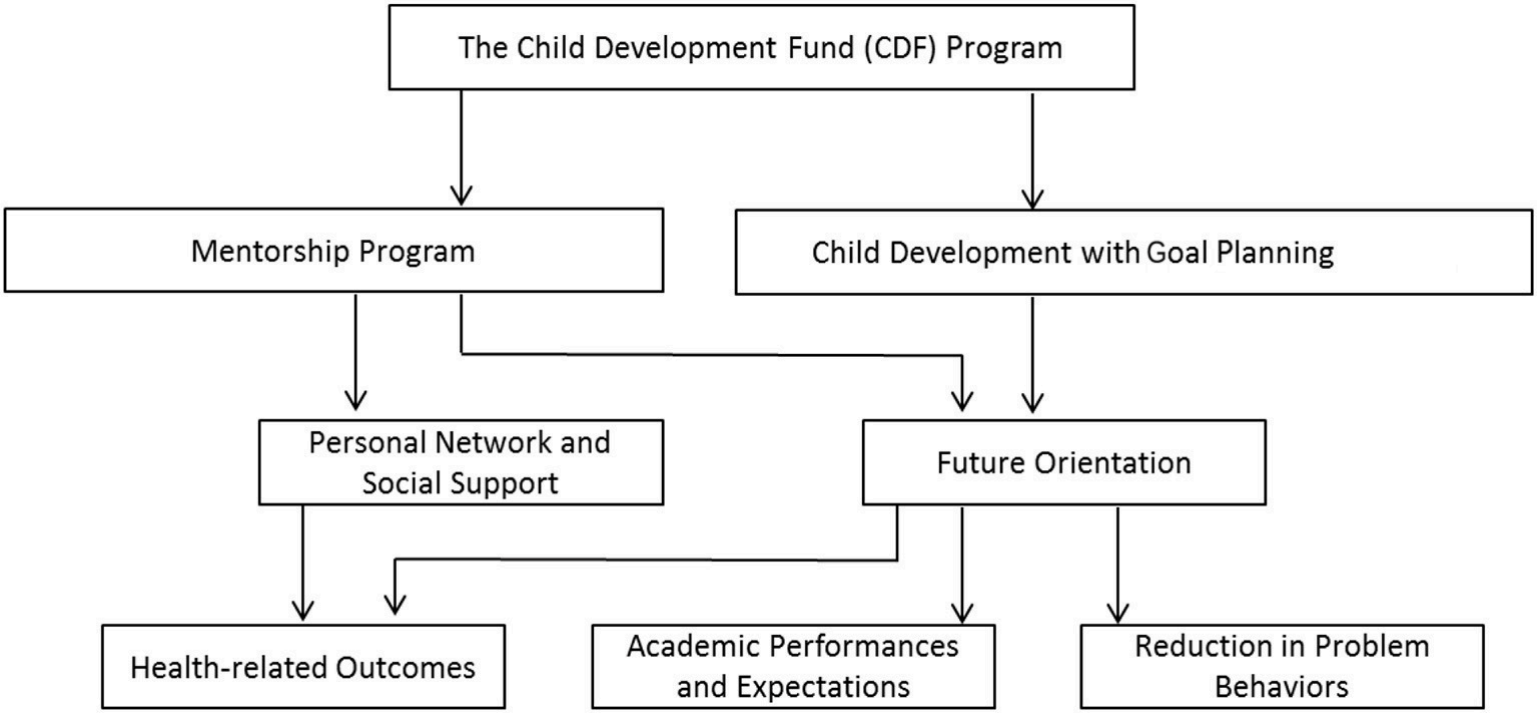
Conceptual framework for the CDF program.

Hong Kong has a significant income disparity with a Gini coefficient of 0.539; 17.2% of children are living in poverty (18, 19). The Child Development Fund (CDF) was established by the government of Hong Kong in 2008 to support non-governmental organizations (NGOs) in regard to rolling out the program. This 3-year community intervention program integrates both CDA and mentoring components and targets socioeconomically disadvantaged children aged between 10 and 16 years. The CDF program aims to help children accumulate financial assets by establishing CDAs, the savings from which will be put toward the children's desired purposes through personal goal planning (Figure 1). Under guidance from NGOs, mentors, and parents, the CDF participants develop their short-term and long-term goals and utilize their accumulated savings to achieve their goals. The CDF is unique in that it also encourages non-financial asset building through a mentorship program involving volunteer mentors (20, 21). The mentors provide guidance and share life experience with participating children, and assist them to build up non-financial assets. A previous evaluative study has shown that children who participated in the CDF program during 2009 and 2011 outperformed non-participants in regard to financial asset development and that positive mentorship was associated with positive psychosocial development (20, 21). To offer additional knowledge to the literature on the longer-term psychosocial characteristics of adolescents who have participated in an asset building program and to extend previous findings in order to further understand the role of mentoring in such programs, this study aims to: (1) compare the CDF participants' characteristics in terms of health, behaviors, attitude, schooling, and social support against a comparison group; and (2) examine factors that moderate the effects of the CDF.

## Methods

### Study participants

The sample consisted of 902 adolescents from low-income families in Hong Kong. Of the adolescents, 552 were CDF participants and 350 formed a comparison group consisting of adolescents who were eligible to but did not participate in the CDF. The CDF participants completed the program between 2011 and 2015. All participants were from families receiving Comprehensive Social Security Assistance, full grants from Student Financial Assistance Schemes, or whose monthly household income was lower than 75% of the median monthly domestic household income, which was equivalent to USD 3205.13 (22). The CDF program has been described in detail elsewhere (23).

### Study design and procedure

This study was an evaluative study of the CDF community intervention and was conducted between January and June 2016. The NGO operators implemented the CDF program approached and invited all 1950 potential subjects (1521 CDF participants and 429 non-participants) with valid contact details (the telephone numbers or home addresses). Consent was secured from 918 of these individuals, including 568 CDF participants, and 350 non-CDF-participants, giving the response rate of 47.1% (37.3% for the CDF participants and 81.6% for the comparison group). The final sample comprised of 552 CDF participants and 350 non-participants who completed the survey. Most of the CDF participants who did not participate in the survey indicated that they were occupied with public examinations or school assignments. The contact information of some of the CDF participants was outdated. Written informed consent was obtained from parents of adolescents under age 18 and written informed consent was obtained from the participants over age 18. The study was approved by the Institutional Review Board of the University of Hong Kong/ Hospital Authority Hong Kong West Cluster.

### Measures

#### Outcome measures

##### Behavioral problems

Four subscales of the Chinese version of the Strengths and Difficulties Questionnaire (SDQ) (24) were used to measure four difficulties (conduct problems, hyperactivity, emotional problems, and peer problems). Each of the four subscales contained five items and all items were rated on a three-point scale. Higher scores indicate higher frequencies of behavioral problems. The Cronbach's alphas for the subscale ranged from 0.45 to 0.76.

##### Perceived social support

The Multidimensional Scale of Perceived Social Support (MSPSS) (25) captures perceived social support from family (four items), friends (four items), and significant others (four items). The items were rated on a seven-point scale. Higher scores indicate higher levels of social support. The Cronbach's alphas for the Chinese MSPSS subscales and the overall score ranged from 0.86 to 0.94 (26).

##### Hope

Participants' future orientations were assessed using the Hope Scale (27) which is a 12-item instrument measuring two components of hope: agency (i.e., goal-directed determination) and pathways (i.e., plans to accomplish goals). Participants responded to each item on a four-point scale. Higher scores indicate higher levels of hope. The Cronbach's alpha for the total score was 0.74 (27).

##### Child health-related quality of life (HRQoL)

The Chinese version of the Pediatric Quality of Life Inventory, Generic Core Scale (Child Version) (28) was used to measure health-related quality of life in regard to physical functioning (eight items), emotional functioning (five items), social functioning (five items), and school functioning (five items). All items were rated on a five-point scale. To ease analysis and interpretation, all item scores were converted in this study to a scale from 0 = *very poor* to 100 = *very good*. Higher scores indicate better HRQoL.

##### Motivation to study

A single item was used to assess participants' motivation to study by rating it on a 10-point scale (from 1 = *I have no interest at all in studying* to 10 = *I have a strong interest in studying*). A higher score indicates a stronger motivation to study.

##### School withdrawal behaviors

Four items were used to measure the number of times the participants were late, absent, truant, and given demerits during the past year (where 1 = *none*, 2 = *1–2 times*, 3 = *3–5 times*, 4 = *6–10 times*, and 5 = *11 times or more*). All items were averaged to obtain an overall score. Higher scores indicate more problems in school.

##### Understanding of academic subjects

articipants rated the extent to which they could understand the contents of three major academic subjects (Chinese language, English language, and mathematics), each on a five-point scale (from 1 = *fully understand* to 5 = *do not understand at all*). A mean score of these items was computed. Higher scores indicate lower levels of understanding of the academic subjects.

#### Potential moderator

##### Mentorship quality

Mentorship quality was measured using eight items rated on a four-point scale (from 1 = *strongly disagree* to 4 = *strongly agree*), regarding questions such as “My mentor tried to understand my development targets, such as academic studies and other personal matters.” Higher scores indicate better mentorship quality.

#### Covariates

The genders, ages, and family incomes of the CDF participants and the comparison group were surveyed.

### Statistical analyses

The participants' demographic characteristics were summarized using descriptive statistics. To test the effects of the intervention on the outcome measures, a series of adjusted regression analyses were performed, with gender, age, and family income included as covariates. For each regression analysis, the Cohen's *d* effect size was computed to indicate the mean difference between the two groups in standard deviation units. A series of moderation analyses were conducted to examine the potential moderating effects of gender and mentorship quality on the intervention. Statistical significance was determined by two-tailed tests, with *p* < 0.05. All analyses were performed using R Statistical Software v3.4.3.

## Results

Table 1 shows the demographic characteristics of the participants. Of the CDF participants, 56.0% were female and 44.0% were male. Their mean age was 18.30 years and the mean monthly family income was USD 1852.63 (*SD* = 988.42). Of the comparison group, 54.6% were female and 45.4% were male. Their mean age was 17.98 years and the mean monthly family income was USD 2020.04 (*SD* = 639.75). The CDF participants and the comparison group differed significantly in regard to family income; therefore, family income was adjusted as a potential confounder in the subsequent analyses. However, the two groups are still comparable, as the family incomes of both groups were significantly lower than the median monthly household income of the population, which is USD 3205.13 (22) (*p* < 0.0001).

**Table 1:** Characteristics of CDF participants and non-participants.

	**Total (*N* = 902)**	**Participants (*n* = 552)**	**Non-participants (*n* = 350)**	***p*-value**
Age	18.17 (2.55)	18.30 (2.37)	17.98 (2.80)	0.07
Gender				0.68
Female	500 (55.4%)	309 (56.0%)	191 (54.6%)	
Male	402 (44.6%)	243 (44.0%)	159 (45.4%)	
Monthly family income (USD)	1,918.82 (877.60)	1,852.63 (998.42)	2,020.04 (639.75)	0.005

As shown in Table 2, the CDF participants had fewer behavioral problems, particularly in regard to emotional (B = −0.61, *p* < 0.001), peer (B = −0.38, *p* < 0.001), and conduct problems (B = 0.79, *p* < 0.001). There was also evidence of the intervention benefiting social support from all sources, including significant others (B = 0.35, *p* < 0.001), family (B = 0.30, *p* < 0.001), and friends (B = 0.41, *p* < 0.001). The intervention may also improve understanding of academic subjects (B = 0.11, *p* < 0.05), motivation to study (B = .37, *p* < 0.01), and school withdrawal behaviors (B = −0.11, *p* < 0.05). Both hope subscales were better for participants than non-participants, but the effect was stronger in the pathway subscale (B = 0.16, *p* < 0.001) than in the agency subscale (B = 0.07, *p* < 0.05). The intervention effect on health-related quality of life was not statistically significant, except for the social functioning subscale (B = 2.45, *p* < 0.05).

**Table 2:** Evaluation of the CDF intervention program.

	**Effect of Participation**	***p*-value**	**Cohen's *d***
**BEHAVIORAL PROBLEMS**			
Emotional problems	−0.61 (−0.91, −0.30)	<0.001	−0.13
Peer problems	−0.38 (−0.58, −0.18)	<0.001	−0.13
Conduct problems	−0.79 (−1.01, −0.56)	<0.001	−0.23
Hyperactivity	−0.26 (−0.52, 0.01)	0.06	−0.06
Total difficulty	−2.03 (−2.75, −1.32)	<0.001	−0.19
**PERCEIVED SOCIAL SUPPORT**			
From significant others	0.35 (0.21, 0.49)	<0.001	0.16
From family	0.30 (0.16, 0.44)	<0.001	0.14
From friends	0.41 (0.27, 0.55)	<0.001	0.19
Total	0.35 (0.22, 0.48)	<0.001	0.18
**SCHOOLING**			
Understanding of academic subjects	0.11 (0.01, 0.22)	0.03	0.07
Motivation to study	0.37 (0.14, 0.60)	0.002	0.10
School withdrawal	−0.11 (−0.19, −0.03)	0.008	−0.09
**HOPE**			
Pathway	0.16 (0.10, 0.22)	<0.001	0.19
Agency	0.07 (0.00, 0.13)	0.04	0.07
Total	0.11 (0.06, 0.17)	<0.001	0.14
**HRQOL**			
Physical	1.56 (−0.39, 3.51)	0.12	0.05
Emotional	−1.44 (−4.13, 1.24)	0.29	−0.04
Social	2.45 (0.19, 4.70)	0.03	0.07
School	1.59 (−0.83, 4.01)	0.20	0.04
Total	1.04 (−0.92, 3.00)	0.30	0.03

*Intervention effects were estimated with control of age, gender, and family income. Cohen's d denotes effect sizes, with 0.2, 0.5, and 0.8 corresponding to small, medium, and large effects, respectively*.

**Figure 2:**
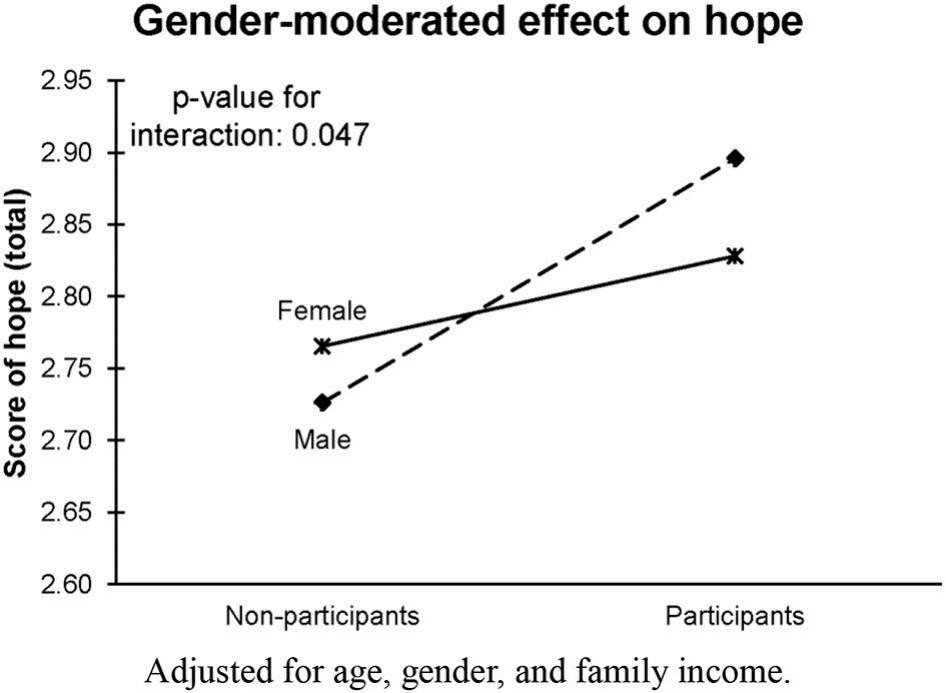
Effect of CDF participation on hope, moderated by gender. Adjusted for age, gender, and family income.

**Figure 3:**
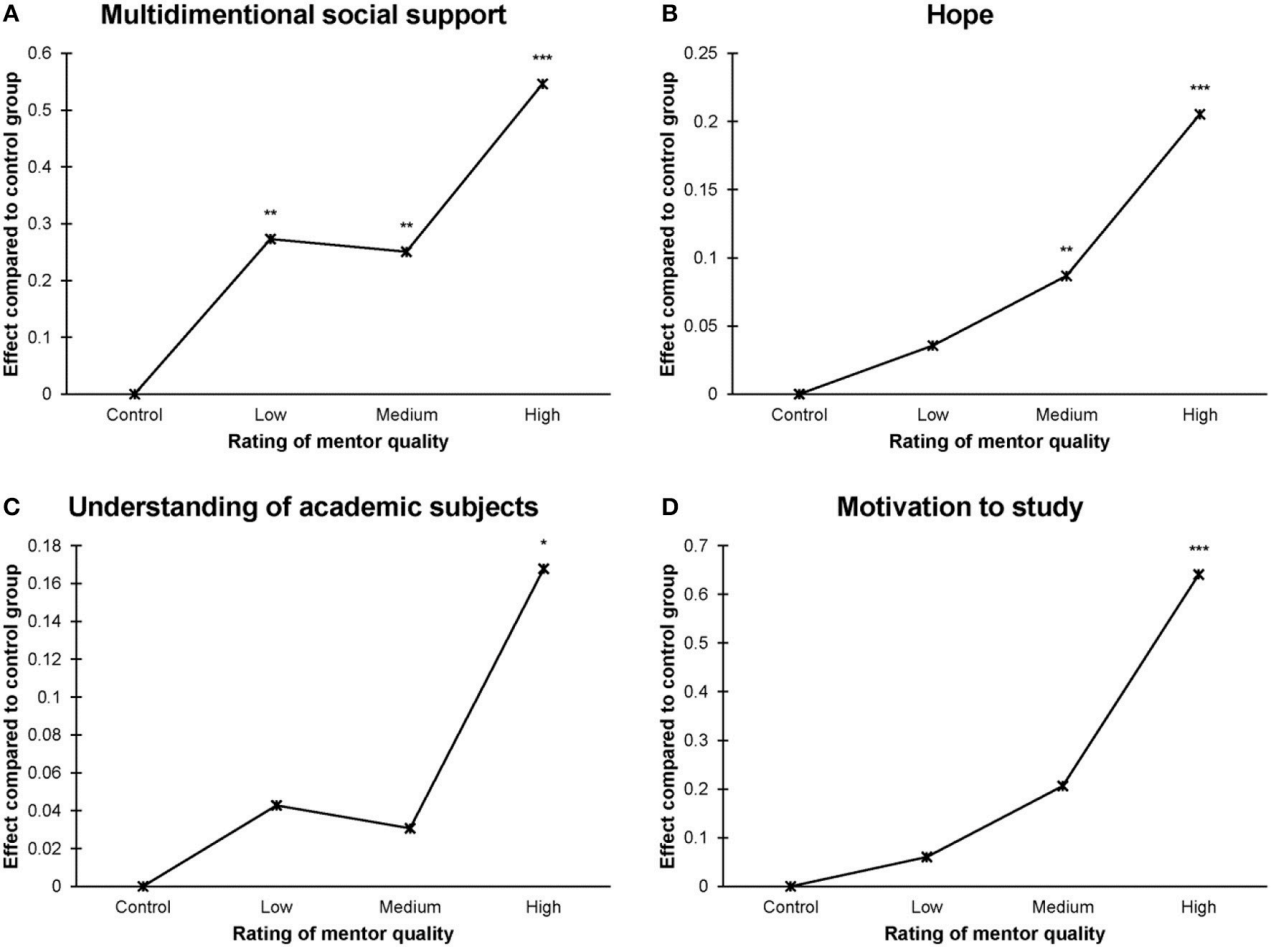
Effects of CDF participation on **(A)** multidimensional social support, **(B)** hope, **(C)** understanding of academic subjects, and **(D)** motivation to study, moderated by prticipants' rated mentor quality. Adjusted for age, gender, and family income. ^***^p < 0.001, ^**^p < 0.01, ^*^p < 0.05.

Figure 2 shows that gender moderated the intervention's effect on hope, with a stronger intervention effect among males than females. Furthermore, there was a moderating effect of mentorship quality on the intervention's effects. Figure 3 shows that participant-rated mentor quality appears to be a strong moderator for the intervention's effectiveness. Perceived social support, hope, understanding of academic subjects, and motivation were improved the most when the participants rated their mentors as being helpful and understanding.

## Discussion

This study contributes to the limited extant literature by demonstrating that the disadvantaged adolescents who participated in an asset-building program may have better outcomes in regard to social functioning, social support, attitudes toward studying and the future, and fewer behavioral problems than the non-participants, despite the differences were small as indicated by Cohen's d effect sizes. Several features of the CDF program in this study may have contributed to these differences. The savings accounts with the personal goal planning component may have enabled adolescents to set their own goals and use their savings to work toward achieving their goals, the process of which may have enhanced the adolescents' hope regarding the future. Future orientation has been found in previous studies to have a positive influence on a range of behaviors, such as academic performance and attainment, and risky behaviors (29–31). Consistent with previous findings (12) this study found that the participating adolescents appeared to have fewer behavioral problems, including social, emotional, and conduct problems. The mentoring component of the program may have expanded adolescents' personal networks for additional support and guidance; hence, adolescents perceived higher levels of social support from others, which in turn enhanced their social functioning. Social support is particularly important for adolescents, as it has long been known to promote adolescent's well-being in various aspects, including academic achievement and psychological adjustment (32). The CDF participants and non-participants did not display significant differences in regard to various dimensions of the HRQoL, which may be due to the fact that the CDF design did not involve an active health intervention component. Rather, the program aimed to strengthen participants' psychosocial development, thereby enhancing their physical health.

Extending previous findings suggesting that good mentorship may affect CDF participants' future planning and self-efficacy in regard to career goal setting (33) this study found that mentorship quality moderated the intervention's effects in regard to adolescents' perceived social support, hope, understanding of academic subjects, and motivation to study. This finding supports the crucial role that mentors play in underprivileged adolescents' lives (17). In addition to this, although both female and male CDF participants benefitted significantly from the intervention in terms of hope, the males derived more from the intervention.

### Implications

The findings of this study can inform the designs of future asset-based programs that aim to ease poverty. This study supports mentorship as a critical component in asset-building programs. Future programs may consider mentoring as a supplemental intervention, as youths with fewer resources are less likely to have mentors but are likely to benefit more from having a mentor, compared to those who are more resourceful (17). Program components that help children improve their health outcomes are necessary. For example, in Hong Kong, public healthcare services are heavily subsidized by the government (34). As parents with low SES may have less health knowledge and fewer desirable health behaviors (35) providing parent's education and connecting these families to public healthcare services may be effective strategies. In terms of policy, the positive findings of this study support the recent policy address by the Hong Kong government regarding the further development of the CDF (36). In research terms, several areas deserve greater attention. Longitudinal tracking of participants into the time at which they enter the workforce would help shed light on the effectiveness of these projects in easing intergenerational poverty. Future studies examining the mediating mechanisms by which financial and non-financial assets affect child outcomes are also needed.

### Limitations

Several limitations of this study should be noted. First, this study lacks longitudinal data. Despite the CDF participants outperforming the non-participants in various psychosocial characteristics, factors other than the CDF program may have contributed to these differences. Second, this study was an evaluation of community intervention (i.e., non-random assignment), which is subject to selection bias and the findings of this study may not be generalizable to other settings. Although a more rigorous study design, such as one involving a randomized controlled trial, would be desirable, adopting such a design for a large-scale community-based project at the governmental level would be difficult and costly. Third, this study was unable to test the CDF's impact on the reduction of intergenerational poverty, which requires a longer-term follow-up study. Finally, this study is challenged by a low response rate in particular for the CDF participants, partly due to some participants were lost during this study's follow-up, which inevitably poses the possibility of non-response bias. Previous community intervention studies have shown response rates between 38 and 69% (10, 37). Despite these limitations, this study attempted to overcome difficulties to assess longer term psychosocial development of a relatively large sample size of adolescents from low socio-economic backgrounds who participated in a community-based poverty alleviation program consisted of CDAs and mentorship components.

## Conclusion

This study provides preliminary positive evidence that the adolescents who participated in the CDF program outperformed the adolescents in the comparison group in regard to behavioral, social, academic, and attitude aspects. The findings also show that higher levels of mentorship quality moderated the effect of the program. These findings can inform policy design to ease poverty.

## Author contributions

KC conceptualized the study design, interpreted the data, and critically revised the manuscript. CL interpreted the data and drafted the manuscript. FH analyzed the data and drafted the manuscript. SZ and SL interpreted the data and critically revised the manuscript. PI assisted in conceptualizing the study design, interpreted the data, and critically revised the manuscript. All authors approved the final manuscript as submitted.

### Conflict of interest statement

The authors declare that the research was conducted in the absence of any commercial or financial relationships that could be construed as a potential conflict of interest.
